# Taxifolin for prevention of COGnitive impairment (T-COG trial): a study protocol for a randomized, double-blind, placebo-controlled trial

**DOI:** 10.3389/fnut.2025.1686381

**Published:** 2025-10-13

**Authors:** Tetsuya Chiba, Yorito Hattori, Koko Asakura, Yuka Sano, Satoshi Saito, Manabu Minami, Haruko Yamamoto, Masafumi Ihara

**Affiliations:** ^1^Department of Neurology, National Cerebral and Cardiovascular Center, Suita, Japan; ^2^Department of Preemptive Medicine for Dementia, National Cerebral and Cardiovascular Center, Suita, Japan; ^3^Department of Data Science, National Cerebral and Cardiovascular Center, Suita, Japan; ^4^Department of Clinical Research Promotion Center, National Cerebral and Cardiovascular Center, Suita, Japan

**Keywords:** taxifolin, Alzheimer’s disease, mild cognitive impairment, randomized control trial, double-blind, placebo

## Abstract

**Background:**

In 2023 and 2024, the novel anti-*β*-amyloid antibodies lecanemab and donanemab have been approved for treatment of mild cognitive impairment and mild dementia in several countries, including Japan and the United States. Although they successfully eliminate accumulated *β*-amyloid, they merely delay cognitive deterioration and do not improve cognitive function. This suggests that *β*-amyloid elimination is insufficient for cognitive improvement. Therefore, novel treatments with pleiotropic neuroprotective effects are warranted. Taxifolin, a bioactive flavonoid, shows pleiotropic effects, such as inhibition of amyloid-*β* aggregation and oligomerization and hippocampal neuroinflammation, as well as stimulation of brain lymphatic vessel formation in our previous experimental studies. Furthermore, our preliminary observational study showed that oral administration of taxifolin was associated with cognitive improvement in patients with mild cognitive impairment or mild dementia.

**Methods:**

This is a randomized, double-blind, placebo-controlled, crossover trial involving 60 patients with mild cognitive impairment or mild dementia. All participants will take 100-mg taxifolin or placebo capsules orally once daily for 12 weeks. The washout period will be 6 weeks. The primary objective is to determine the effect of taxifolin on cognitive impairment using the Montreal Cognitive Assessment. The main secondary objectives are to evaluate the impact of taxifolin on (i) prevention further cognitive decline, as evaluated by changes in the scores for total Alzheimer’s Disease Assessment Scale-Cognitive Subscale 14 and trail making test and (ii) changes in white matter hyperintensity volume and number of cerebral microbleeds on brain magnetic resonance imaging.

**Discussion:**

This T-COG trial may provide valuable insights into new therapeutic approaches, considering that taxifolin has multitarget neuroprotection, which could prevent further cognitive decline, along with its highly safe profile and inexpensive cost.

**Clinical trial registration:**

https://jrct.mhlw.go.jp, jRCTs051250004.

## 1 Introduction

The number of people with dementia exceeded 57.4 million in 2019 and is estimated to reach approximately 152.8 million by 2050 worldwide (1). Early in Alzheimer’s disease (AD) pathogenesis, *β*-amyloid deposition in the brain promotes senile plaque formation and cerebral amyloid angiopathy, while subsequent accumulation of phosphorylated tau drives neurofibrillary tangle formation (2, 3). *β*-amyloid peptides, generated by the sequential cleavage of amyloid precursor protein by β- and *γ*-secretases, initially form oligomers. These *β*-amyloid oligomers exert neurotoxic effects by altering synaptic morphology and density, thereby impairing synaptic plasticity independently of mature *β*-amyloid fibrils. Subsequently, oligomers aggregate into protofibrils and cross-linked *β*-sheet fibrils, ultimately forming amyloid plaques. β-amyloid aggregates also interact with tau protein and contribute to neurotoxicity. Collectively, β-amyloid fibrils and plaques, together with neurofibrillary tangles, drive additional pathological processes in AD, including oxidative stress, neuroinflammation, and mitochondrial dysfunction, leading to neuronal dysfunction and cell death (3). Based on the aforementioned amyloid hypothesis, novel treatments have been developed to target and inhibit *β*-amyloid accumulation. Currently, the humanized IgG monoclonal antibodies lecanemab and donanemab are approved as novel disease-modifying drugs for early AD by degrading protofibril *β*-amyloid and pyroglutamate-modified β-amyloid, respectively (4, 5). However, lecanemab and donanemab have the following weaknesses that must be overcome (4, 5): (i) mere suppression of cognitive deterioration and not improvement of cognitive function, despite successful elimination of accumulated *β*-amyloid; (ii) may lead to neurological disorders and serious or life-threatening events that present as amyloid-related imaging abnormalities, including brain edema, and cerebral microbleeds or hemorrhage; and (iii) expensive cost ($26,500 or $32,000 per patient per year in the United States) (6, 7), although these therapies may be widely accessible. Considering that treatments that target *β*-amyloid alone is likely insufficient, pleiotropic neuroprotective effects may be required to achieve cognitive improvement or prevent cognitive deterioration.

Taxifolin (dihydroquercetin) is a natural bioactive flavonoid that can be isolated from grapes, citrus fruits, onions, green tea, olive oil, wine, and several herbs. It is a potentially useful treatment for cardiovascular diseases (8); malignant tumors, such as breast cancer (9); COVID-19 (10); diabetes (11); and obesity (12). Mechanistically, taxifolin has been suggested to exhibit antiinflammatory, antioxidant, anticancer, antitumor, antiapoptotic, and mitochondrial protective effects (13). Furthermore, we demonstrated its therapeutic effects against AD and cerebral amyloid angiopathy (5). Our experimental studies showed that taxifolin led to inhibition of *β*-amyloid 40 and β-amyloid 42 aggregation (14, 15), oligomer formation (14), and hippocampal neuroinflammation (16) and stimulation of brain lymphangiogenesis (16). Our preliminary observational study without placebo successfully showed that oral taxifolin intake was associated with cognitive improvement which was assessed by the Montreal Cognitive Assessment (MoCA), and no adverse events among patients with mild cognitive impairment or mild dementia (17). Therefore, owing to its pleiotropic neuroprotective effects, taxifolin has the potential to prevent further cognitive decline.

We designed this randomized, double-blind, placebo-controlled clinical trial named “Taxifolin for prevention of COGnitive impairment (T-COG trial)” to investigate the efficacy of taxifolin in preventing further cognitive decline in patients with mild cognitive impairment or mild dementia.

## 2 Methods and analysis

### 2.1 Trial design

**Figure 1:**
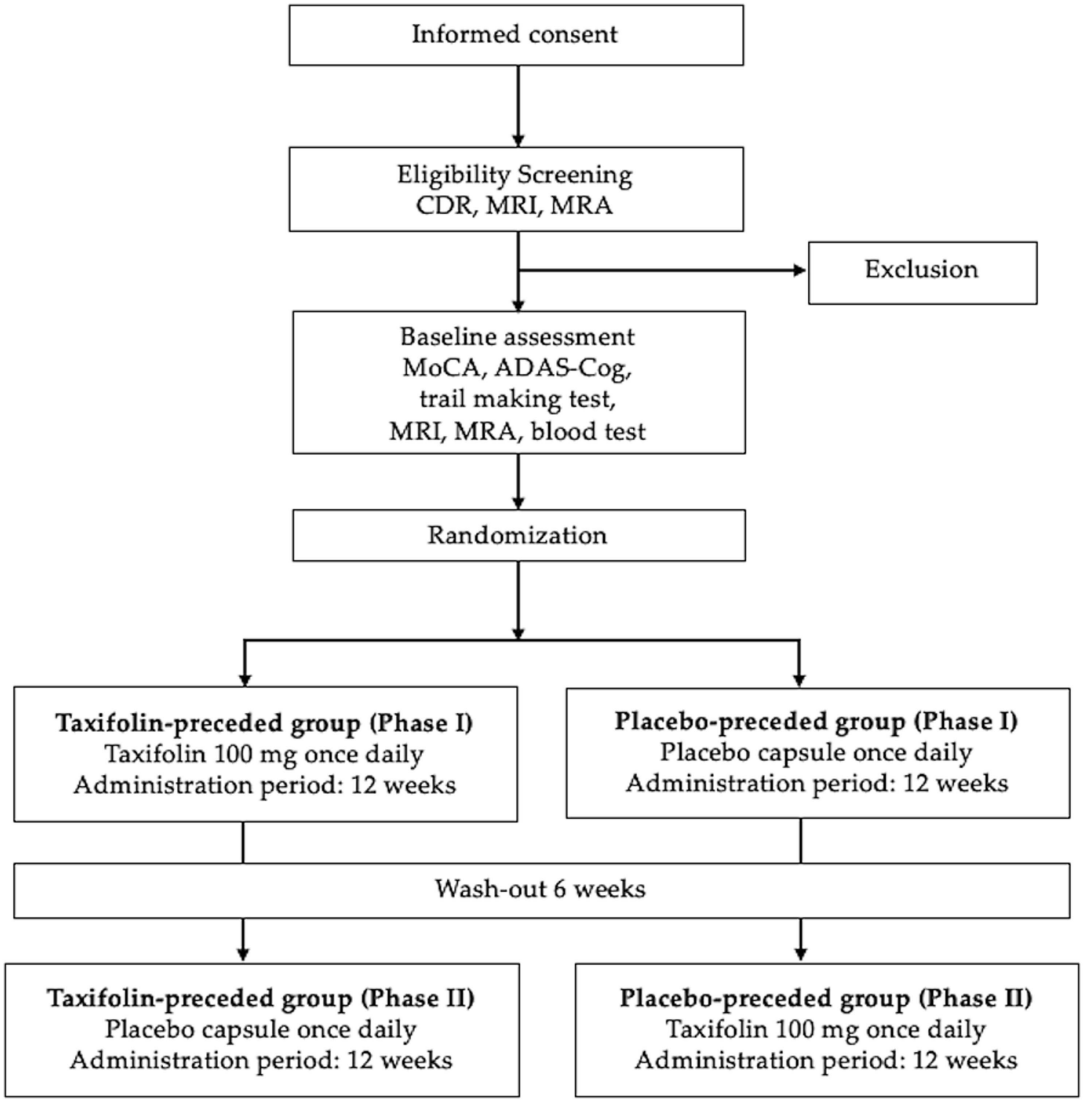
Schematic flow diagram of the participant enrollment process in the T-COG trial. ADAS-Cog, Alzheimer’s disease assessment scale-cognitive subscale 14; CDR, clinical dementia rating; MoCA, Montreal Cognitive Assessment; MRA, magnetic resonance angiography; MRI, magnetic resonance imaging.

The T-COG trial will be a randomized, double-blind, placebo-controlled, crossover trial with washout and will be performed at the National Cerebral and Cardiovascular Center (NCVC). It was approved by the NCVC Clinical Research Review Board (approval number: NCVC-CRB2501) and was prospectively registered in the Japan Registry of Clinical Trials (registration number: jRCTs051250004) on April 7, 2025. This trial will be conducted according to the principles of the Helsinki Declaration and the Clinical Trial Act in Japan. Patients will be asked to provide written informed consent to participate in this trial. Using 1:1 ratio, 60 patients will be randomly assigned to receive oral 100-mg taxifolin capsules or matching placebo capsules once daily for the first 12 weeks (phase I), followed by a 6-week washout period, then a second 12-week treatment period with the alternative therapy (phase II) (Figure 1).

The physicians involved will provide sufficient information on this clinical trial and the NCVC Biobank, and patients will be given adequate time to decide on participation in this clinical trial. If a patient decides to participate voluntarily, written informed consent will be obtained. In case of any revision in the written informed consent form, the physicians involved in the trial will again explain the revised procedure to the patients, revise the written informed consent form, and obtain each patient’s voluntary consent to continue participation. As a dissemination policy, the research findings after the trial will be submitted for publication in a peer-reviewed journal.

### 2.2 Study objectives

To assess the effectiveness of taxifolin in preventing further cognitive decline in patients with mild cognitive impairment or mild dementia, the primary objective will be difference (*Δ*) in the MoCA total score from baseline. The principal secondary objectives will be to evaluate the impact of taxifolin on (i) prevention further cognitive decline, as evaluated by changes in the scores for total Alzheimer’s Disease Assessment Scale-Cognitive Subscale 14 (ADAS-Cog) and trail making test from baseline and (ii) changes in white matter hyperintensity volume and number of cerebral microbleeds on brain magnetic resonance imaging (MRI) (Table 1).

**Table 1:** Primary and secondary objectives.

Primary objective
Difference (Δ) in the MoCA total score from baseline to 12 weeks
Secondary objectives
(1) Presence or absence of changes in the MoCA total score from baseline to 12 weeks (2) Changes in the ADAS-Cog total score from baseline to 12 weeks. (3) Changes in completion time on the trail making test part B from baseline to 12 weeks. (4) Changes in cerebral white matter lesions (Fazekas classification and volume) on brain fluid-attenuated inversion recovery -MRI from baseline to 12 weeks. (5) Changes in the number of cerebral microbleeds on brain MRI from baseline to 12 weeks. (6) Change in body weight (kg) from baseline to 12 weeks.

ADAS-Cog, Alzheimer’s disease assessment scale-cognitive subscale 14; MoCA, Montreal cognitive assessment; MRI, magnetic resonance imaging.

### 2.3 Inclusion criteria

The T-COG trial will be conducted by investigators at the NCVC. We will include patients in the following categories: (i) aged ≥40 to <90 years; (ii) with a clinical dementia rating (CDR) global score of 0.5 or 1.0; and (iii) no history of symptomatic cerebral infarction/hemorrhage, acute cerebral infarction/hemorrhage, brain tumor, severe stenosis or occlusion of the middle cerebral artery, and other brain diseases on brain MRI or MR angiography within 48 weeks prior to obtaining written informed consent or at the time of screening (Table 2).

**Table 2:** Eligibility criteria.

Inclusion criteria
(1) Patients aged ≥40 to <90 years; (2) Patients with a clinical dementia rating global score of 0.5 or 1.0; (3) Patients who do not have history of symptomatic cerebral infarction/hemorrhage, acute cerebral infarction/hemorrhage, brain tumor, severe stenosis or occlusion of the middle cerebral artery, and other brain diseases on brain MRI or MRA within 48 weeks prior to obtaining written informed consent or at the time of screening; and (4) Patients or their family members provided written informed consent.
Exclusion criteria
(1) Patients who cannot undergo neuropsychological examinations and brain MRI; (2) Patients who have not been diagnosed with secondary cognitive impairment, such as Parkinson’s disease, Parkinson’s syndrome, Huntington’s disease, normal pressure hydrocephalus, progressive supranuclear palsy, multiple system atrophy, multiple sclerosis, head injury with residual sequelae, neurosyphilis, hypothyroidism, vitamin B1/12 deficiency, and folate deficiency; (3) Patients who do not cease the intake of taxifolin at least 6 weeks prior to the administration of the experimental drugs, if these patients were already on taxifolin; (4) Patients currently receiving lecanemab or donanemab; (5) Patients in whom the dosage or administration of an antidementia drug, such as donepezil, galantamine hydrobromide, rivastigmine, and memantine hydrochloride, is changed within 4 weeks prior to providing their informed consent; (6) Patients with past history of intrinsic psychiatric disease or alcohol or drug dependence within 48 weeks before giving their informed consent; (7) Patients with severe systemic diseases, such as heart failure, liver failure, kidney failure, and hypothyroidism; (8) Patients with a gelatin allergy; (9) Patients diagnosed with malignancy within the past 5 years; (10) Pregnant and breastfeeding women or patients who do not agree to use appropriate contraception; and (11) Patients participating or planning to participate in other clinical trials using other medicines or medical devices.

MRA, magnetic resonance angiography; MRI, magnetic resonance imaging.

### 2.4 Screening procedures

Prior to enrollment, eligibility of all potential participants will be determined by a screening process, which includes review of medical records and cognitive testing results, including CDR. To protect confidentiality before, during, and after the study, the personal information of potential and enrolled patients will be shared in a database that is accessible by only those within the project group responsible for patient enrollment. At the first visit, the physician involved in the trial will provide the patient and/or his/her family with detailed information about the T-COG trial and obtain written informed consent. The consent form will state that participants have the right to withdraw from the study without any disadvantage. The physician in charge will perform a physical examination of all participants, and the clinical research coordinator (CRC) will collect the necessary clinical data, including height, weight, vital signs, medical history, supplements, and demographics.

### 2.5 Baseline assessments

**Figure 2:**
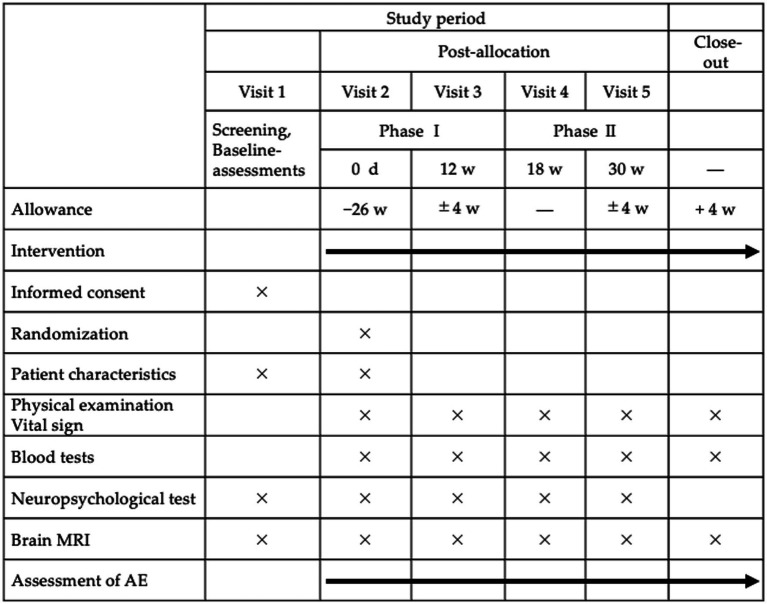
Schedule of interventions and assessments in the T-COG trial. Participants will be randomly assigned in a 1:1 ratio to receive taxifolin 100 mg or matching placebo daily for the first 12 weeks (phase I), followed by a 6-week washout period, then a second 12-week treatment period with the alternative therapy (phase II). MRI, magnetic resonance imaging; AE, adverse events.

After initial screening, all participants will undergo a neuropsychological assessment using the MoCA, ADAS-Cog, and trail making test. Brain MRI will be performed during the second visit. Blood samples for analysis will be collected before supplementation of taxifolin or placebo capsules (Figure 2). The physicians involved in the trial will distribute the capsules to the participants after randomization (see section on randomization, allocation, and blinding). To ensure proper medication compliance, all participants will be instructed to fill in a daily schedule book for the T-COG trial and to return any extra capsules during each visit.

### 2.6 Follow-up assessments

Phase I will end at 12 weeks and be followed by a 6-week washout period; phase II will be between weeks 18 and 30. All participants will be required to visit the NCVC at weeks 12, 18, and 30 for neuropsychological assessment, brain MRI, and blood tests. In addition, each physician involved in the trial will perform physical examinations, and the CRC will conduct interviews about changes in prescription drugs, over-the-counter drugs, and supplements, as well as occurrence of adverse events. To facilitate participant retention and follow-up, the CRC will call the patients at least once a month to check on continued intake of taxifolin or placebo capsules.

### 2.7 Randomization, allocation, and blinding

The physicians involved in the trial will generate the allocation sequence, enroll patients, and assign patients to the interventions. The registered patients will be assigned randomly to either the taxifolin- or placebo-preceded group. The assignment method will be minimization, and the stratification factors considered will be age, sex, and CDR global score of 0.5/1. The randomization process will be executed using computer-generated random allocation treatment codes. Both patients and physicians will remain blinded to the assigned therapy throughout the study. The randomization list will be maintained by an independent investigator who is not involved in patient care, assessment, data collection, or analysis. Emergency unblinding will occur only if the principal investigator deems it necessary to reveal the assigned intervention to manage any potential adverse events in the patients.

### 2.8 Interventions

Standardized taxifolin and placebo capsules will be supplied by Towa Pharmaceutical Co., Ltd., Japan. The manufacture of taxifolin capsules will use 99% pure synthetic taxifolin (Lavitol™, DHQ, Saitama, Japan) and comply with Good Manufacturing Practice. The appearance of the placebo capsules is identical to that of the taxifolin capsules. The detailed ingredients of the placebo capsules are shown in Table 3. Participants will take with water one 100-mg taxifolin capsule or one placebo capsule once daily for 12 weeks. The physicians or CRCs involved in the trial will maintain detailed capsule inventory records to document the supply, receipt, disposal, and return of the study capsules.

**Table 3:** The detailed ingredients of the taxifolin and placebo capsule.

Ingredients	Ingredients (mg/capsule)
Starch	211.75
Gelatin	77
Calcium stearate	4.5
Silicon dioxide	3.75
Total	297

The discontinuation criteria will comprise one of the following: (i) severe systemic diseases, such as heart failure, liver failure, renal failure, hypothyroidism, and cancer or (ii) initiation of antidementia medication, such as lecanemab, donanemab, donepezil, galantamine, rivastigmine, and memantine. A restricted medication will be allowed if the doses of antidementia drugs, such as donepezil, galantamine, rivastigmine, and memantine, and calcium channel blockers are unchanged during the trial. This is based on our previous preliminary observational study, which showed that calcium channel blockers were associated with the interval of positive change in the total MoCA score (6). Furthermore, calcium channel blockers have been suggested to improve cerebral blood flow and reduce intracellular calcium load caused by *β*-amyloid binding to presynaptic (P/Q-type) or postsynaptic (L-type) calcium channels (18).

### 2.9 Neuropsychological examination

Neuopsychological examinations, including ADAS-Cog, MoCA, and trail making test, can be performed only by psychologists who have sufficient experience and will be blinded to the intervention groups.

### 2.10 Sample size estimates

This study is exploratory, and sufficient prior data are not available due to differences in the taxifolin dose and observation period, compared with those in our preliminary observational study. Therefore, considering feasibility, the total sample size was pragmatically set at 60 participants (30 in each sequence group). Assuming a 20% dropout rate, the number of participants available for analysis was estimated to be 48.

The primary analysis of our preliminary observational study (17) observed a within-patient difference of 2.1 points in MoCA total score with a standard deviation (SD) of 4.0. Similarly, sensitivity analysis showed a mean difference of 2.6 points with an SD of 4.4. Under the assumption of a similar treatment effect with that in the previous study, a paired t-test with two-sided significance level of 5% would have 94.5% power to detect a mean difference of 2.1 points (SD 4.0) using 48 analyzable participants. Alternatively, under a more conservative scenario, in which the mean difference is 1.6 points (SD 4.0), the statistical power would be 77.5%. All power calculations were conducted using PASS 2024, Version 24.0.2 (NCSS, LLC, Kaysville, UT, United States).

### 2.11 Data collection

A web-based electronic data capture system with secure and restricted access will be used to collect clinical data obtained from patient medical records. Geographic data, medical history, medication information, laboratory data, and findings of neuropsychological tests will be stored in the system. Data will be deidentified only for analysis after this study. Shido Co., Ltd. will be the contract research organization assigned to data management and quality control at each step of data handling, in order to ensure reliability of all trial-related data and maintain the web-based electronic data capture system.

### 2.12 Data monitoring

The individuals assigned to data monitoring will be responsible for safeguarding the interests of the participants, evaluating the safety and efficacy of the interventions throughout the trial, and monitoring the overall conduct of the trial. A formal data monitoring committee will not be established because of the following reasons: (a) long-term administration of taxifolin has minimal risk (17); (b) interim analyses will not be planned; and (c) vulnerable participants who cannot undergo neuropsychological testing (e.g., pediatric patients and those with severe intellectual disability or dementia) will be excluded.

### 2.13 Statistical analysis

Efficacy analyses will be conducted on the analysis population with the intention-to-treat principle. Participants with major protocol violations that may affect efficacy evaluation will be excluded from the per-protocol set; these participants may be included for secondary analyses as needed. The safety analysis set will include all participants who received at least one dose of the study drug (taxifolin or placebo).

The primary endpoint, which is difference (*Δ*) in the MoCA total score from baseline to 12 weeks, will be assessed by assuming a linear mixed-effects model that accounts for period effects. For each sequence group and period, the mean and SD of the change will be calculated. The average ± SD treatment effect will be calculated based on within-patient differences. The overall treatment effect accounting for period effects will be measured using point estimates and confidence intervals. A two-sided paired *t*-test with a 5% significance level will be performed to assess the statistical significance of the overall treatment effect.

For the secondary endpoint, improvement in the MoCA total score will be evaluated using conditional odds ratio, corresponding to McNemar’s test. The change in the ADAS-Cog total score, the change in completion time on the trail making test part B, the change in cerebral white matter lesions (Fazekas classification, volume) on brain fluid attenuated inversion recovery-MRI, the change in the number of cerebral microbleeds on brain MRI, and the change in body weight (kg) from baseline to 12 weeks will be analyzed in a manner similar to the primary endpoint. They will be assessed by assuming a linear mixed-effects model that accounts for period effects. The average treatment effect and its standard deviation will be computed based on the within-patient differences. The overall treatment effect accounting for period effects will be estimated using point estimates and confidence intervals.

Statistical analysis will be performed using SAS version 9.4. (or higher version; SAS Institute Inc., Cary, NC, United States) or R version 4.4.1. (or higher version; R Foundation for Statistical Computing, Vienna, Austria).

### 2.14 Exploratory analysis

An exploratory analysis will be conducted to evaluate temporal changes in plasma inflammatory protein concentrations using a protein biomarker panel (Olink^®^ Target 96 Inflammation; APRO Science group, Tokushima, Japan) for a broad range of studies in which inflammation may play a key role.

### 2.15 Safety profile

If an adverse event occurs, the physicians involved in the trial will treat the patient appropriately in terms of safety and report the details of the event. If a severe adverse event that may be associated with the experimental drugs occurs, the principal investigator must report it to the Director General of the NCVC and the NCVC Clinical Research Review Board. If the adverse event is caused by the protocol treatment, follow-up and best medical treatment will be performed. The patient will be compensated by insurance for any study-related injuries.

## 3 Discussion

Taxifolin holds promise as a novel treatment for cognitive impairment due to mild cognitive impairment and mild dementia, and its potential mechanisms primarily center around inhibition of *β*-amyloid 40 and β-amyloid 42 aggregation (14, 15), oligomer formation (14) and hippocampal neuroinflammation (16) and stimulation of brain lymphangiogenesis (16). Several *in vitro* studies have demonstrated that taxifolin inhibits fibril formation of *β*-amyloid 40 and β-amyloid 42 (14, 15, 19). This may be attributed to the chemical structural characteristics of taxifolin, which is oxidized at the B ring to form an o-quinone structure. The o-quinone structure in the B ring of taxifolin lysine (Lys) 16 and Lys 28 residues, thereby, inhibiting *β*-amyloid 42 fibril formation (16, 19). The degradation induced by taxifolin treatment has been confirmed in *in vivo* studies. In our previous report, we administered taxifolin to cerebral amyloid angiopathy mice and found on filter trap assays that taxifolin may inhibit the formation of β-amyloid oligomers from the monomer (14). Furthermore, in cerebral amyloid angiopathy mice treated with taxifolin, blood β-amyloid levels increased, and promotion of β-amyloid clearance from the brain to the blood was suggested. Moreover, taxifolin suppresses inflammatory changes and oxidative stress in the hippocampus and cerebral cortex, alleviates accumulation of triggering receptors expressed on myeloid cell 2, and reduces glutamate levels. Triggering receptors expressed on myeloid cell 2 is a membrane-spanning protein that is expressed exclusively on brain microglia and has been implicated in the pathology of neuroinflammation-associated neurodegenerative diseases, such as AD and tau pathology, according to several *in vivo* studies (20, 21). Taxifolin inhibits inflammation, oxidative tissue damage, and apoptosis and reduces accumulation of Triggering receptors expressed on myeloid cell 2 -expressing cells (16). Finally, taxifolin may stimulate lymphatic vessel neogenesis in the brain. We previously reported that taxifolin increases the expression levels of lymphatic vessel endothelial hyaluronan receptor 1, a lymphatic vessel neogenesis factor, and vascular endothelial growth factor-D, thereby, potentially promoting the perivascular arterial drainage system for waste removal from the brain (16). Furthermore, increased lymphatic vessel endothelial hyaluronan receptor 1, expression may activate the meningeal lymphatic system, which functions as a drainage pathway from the cerebrospinal fluid to the peripheral blood and may also play a role in regulating the glymphatic system (22).

In this trial, we will be using taxifolin at a dose of 100 mg per day, which is the approved maximum daily intake of taxifolin for adults [Regulation (EC) No 258/97 of the European Parliament and of the Council of January 27, 1997 concerning novel foods and novel food ingredients; and Commission Implementing Decision (EU) 2017/2079 of 10 November 2017 authorising the placing on the market of taxifolin-rich extract as a novel food ingredient under Regulation (EC) No 258/97 of the European Parliament and of the Council]. Therefore, the recommended daily intake of taxifolin at 100 mg is considered appropriate and safe.

In previous crossover studies, the washout period of donepezil was 6 weeks (23). The blood half-life of donepezil is 70 h, whereas that of taxifolin is 8.9 h (24). Therefore, the 6-week washout period in this study is considered reasonable.

In conclusion, the results of the T-COG trial may prove the potential of taxifolin to prevent further cognitive decline in patients with mild cognitive impairment or mild dementia. Furthermore, because taxifolin is derived from natural flavonoids, it is considered safe and affordable. The findings of this trial may provide insights into new treatment approaches for these patients.
